# Patient and Public Involvement Reporting Consistency: Analysis of 24 Clinical Practice Guidelines From 4 Countries

**DOI:** 10.1002/gin2.70061

**Published:** 2026-02-04

**Authors:** Elizabeth Ann Bryant, Rae Thomas, Sharon Lea Sanders

**Affiliations:** ^1^ Institute for Evidence‐Based Healthcare Bond University Robina Queensland Australia; ^2^ Tropical Australian Academic Health Centre Limited (TAAHC) Townsville Queensland Australia

**Keywords:** patient participation, patient preference, Practice Guidelines as Topic

## Abstract

**Objective:**

We compared the reporting of patient and public involvement (PPI) in the development of clinical practice guidelines (CPG) with the guidance regarding PPI in the auspicing organisation's guideline development manual.

**Methods:**

We examined the PPI guidance provided by seven guideline auspicing organisations from four countries, that provided guideline development manuals referencing PPI inclusion in guideline development. We determined whether the auspicing organisations provided guidance on four aspects of PPI critical to understanding the process; (i) which patients and public (PPs) to involve, (ii) how to recruit PPs, (iii) where in the process to involve PPs, and (iv) how PPs should contribute to guideline development. We assessed a random selection of 24 CPGs affiliated with these organisations for consistency of reporting of PPI with the guidance provided.

**Results:**

Half (50%) of the auspicing organisations' guideline development manuals provided guidance on all four aspects of PPI. Five of the 24 CPGs (21%) affiliated with auspicing organisations that made reference to PPI in guideline development did not report PPI in their development. Although 79% (19/24) of guidelines did report PPI in their development, only half (10/19) reported PPI that was consistent with the auspicing organisations guidance.

**Conclusion:**

Auspicing organisations often do not provide guidance to guideline developers on critical aspects of PPI. Even when auspicing organisations do provide guidance, CPG developers may not report PPI, and/or not report PPI consistent with that guidance. It is unknown whether this is because PPI was not conducted, or because it was conducted, but not reported, or was reported incompletely.

**Practice Implications:**

Auspicing organisations need to improve guidance on PPI in guideline development, and CPG developers need to report PPI more diligently when it is done. Understanding guideline developers' motivations for PPI implementation and/or reporting is critical to facilitate improvement in this area.

## Summary

- We examined guideline auspicing organisations recommendations or requirements on public and patient involvement in guideline development.
- We compared the reporting of public and patient involvement in the development of guidelines with these recommendations.
- Few auspicing organisations provide recommendations on all critical aspects of public and patient involvement: who to involve, from where, when and how to involve.
- Clinical practice guidelines may not report public and patient involvement even when recommended or required by guideline auspicing organisations.
- Clinical practice guidelines may not report public and patient involvement consistently with recommendations provided.

## 1 Introduction

International guideline developer organisations endorse patient and public involvement (PPI) as a core principle for developing high‐quality, evidence‐based clinical practice guidelines (CPGs) [1, 2, 3, 4]. Including PPI in CPG development is considered integral for both identifying priority guideline topics [5, 6] and incorporating the values and perspectives of stakeholders in activities that affect them [7, 8]. These inclusions are expected to result in the development of more patient‐centred and trustworthy guidelines [9].

However, there is limited guidance on *how* to incorporate patients and public in guideline development from guideline development organisations [10] or the empirical literature [7, 11, 12]. This may explain, at least in part, the paucity of PPI process reported in CPGs from Australia [1, 13], the United States [14, 15], or Germany [16]. Whether this is an issue solely of reporting and/or a failure to adopt PPI in the development process is unclear. Although reporting guidelines for CPGs include PPI items [17, 18, 19], these items do not currently facilitate the detailed and comprehensive level of PPI reporting necessary to fully understand how PPI occurred or to allow others to replicate it if desired.

When patients and the public *are* involved in guideline development, the process by which this occurs is not well reported. In an examination of 47 studies describing the involvement of patients and publics in guideline development, there was insufficient reporting of information considered basic to an understanding of PPI. This information related to recruiting practices, approaches to inclusion, and when and how to integrate the views of patients and publics [6, 11, 12, 20].

In this study, we aimed to investigate PPI reporting in published CPGs that are auspiced by organisations that recommend and provide guidance for PPI in CPG development. We examined the guidance for, and reporting of, critical PPI information including i) who are the patients and public involved in developing the guideline? ii) from where, and how the patients and public are recruited? iii) at what stage in the guideline development process are the patients and public involved? and iv) how do the patients and public contribute their views? In addition, we assessed whether the CPG developers reported PPI consistent with the recommendations or requirements in the auspicing organisations guideline development manual.

## 2 Methods

This study was conducted in accordance with a protocol developed prior to study commencement. In the absence of a published reporting checklist specifically for methodological studies [21], this study is reported according to the Preferred Reporting Items for Systematic Reviews and Meta‐Analyses extension for Scoping Reviews (PRISMA‐ScR) [22].

We reviewed a random selection of CPGs held in the Guidelines International Network (GIN) library and authored by auspicing organisations from Australia, UK, Canada, and US providing guidance on PPI in the development of CPGs. The CPG was compared with the corresponding auspicing organisation's CPG development manual for PPI reporting consistency. Using the example of the CPG titled “Clinical Practice Guideline: Otitis Media with Effusion (Update)” [23] we provide definitions of the terminology we use in our study in Appendix Table A.1.

### 2.1 Inclusion Criteria

We included clinical practice guidelines if;

1. The CPG was registered in GIN Library and affiliated with an auspicing organisation from Australia, UK, Canada, or the US.
2. The CPG was affiliated with an auspicing organisation that had published a guideline development manual referencing PPI in guideline development.
3. The CPG was published at least 2 years after the auspicing organisation's guideline development manual (allowing for time to implement the PPI framework specified in the manual).
4. The CPG was classified as ‘published’ not ‘living’ (to avoid amendments to the CPG during conduct of this study.

### 2.2 Screening

One study author (EAB) identified the CPGs for inclusion in this review between December 2023 and March 2024 using the following steps.

Step 1: We identified the two most prolific auspicing organisations from Australia, UK, Canada, and the US by using the GIN Library (https://g-i-n.net/international-guidelines-library) search filter ‘Country of Application’. The auspicing organisations with the first and second largest number of CPGs were selected. We then used the ‘Guideline Publication Status’ filter to identify only published CPGs.

Step 2: For the most prolifically publishing auspicing organisations, we sourced and checked their most recent guideline development manual. If the guideline development manual made reference to PPI in CPG development, the auspicing organisation was included for further review. If PPI was not referred to, the auspicing organisation was excluded and the next most prolific auspicing organisation was selected and Step 2 was repeated.

Step 3: We retrieved all CPGs published by the selected auspicing organisations and identified CPGs published at least 2 years after the guideline development manual referencing PPI was published (known as baseline year). This strategy allowed time for authors of the CPGs to incorporate the most recent guideline development manual recommendations.

Step 4: We randomly selected five CPGs from the auspicing organisations that were identified following steps 1–3 using a Random Number Generator (www.calculator.net/random-number-generator.html). If the auspicing organisation had less than five CPGs stored in the GIN Library, then all CPGs from that organisation were included.

### 2.3 Data Extraction

For each countries guideline auspicing organisation, we extracted the guideline development manual title, and the guideline development manual's guidance related to four questions (i) who were the patients and public involved in developing the CPG; (ii) from where, and how the patients and public were recruited; (iii) at what stage in the CPG development process were the patients and public involved, and (iv) how did the patients and public contribute their views. We considered the information obtained from these questions to be basic for understanding and replicating PPI. We developed these questions after discussion with colleagues experienced in guideline development, consideration of the literature related to PPI in clinical practice guidelines and health research, and examination of studies describing the involvement of patients and publics in guideline development [6, 11, 12, 20]. Based on this process we determined that information on which PPI to involve, recruiting practices, approaches to inclusion, and when and how to integrate the views of patients and publics was the minimum essential information needed to evaluate the transparency, inclusivity, and replicability of patient and public involvement in guideline development.

For each CPG developed by the auspicing organization, we extracted the CPG title, and text related to each of the four questions (and the page number where the information appears) when it is reported. We also extracted whether the guideline author included a GIN Guideline Standards Reporting Form (Standards Reporting Form) when submitting a guideline for inclusion in the GIN Library. The voluntary Standards Reporting Form was developed in 2013 [24] following publication of a proposed set of key components for guideline development aiming to promote eventual agreement on a set of international standards for developing CPGs.

The data extraction template was designed by a study author, (EAB) and then piloted independently by the study author and a researcher external to the study team on two randomly selected guideline development manuals and five CPGs. The extractions were discussed, the data extraction form was modified and guidance for completing data extraction developed by the review team (EAB, SS, RT). Using the modified form and guidance, the reviewer (EAB) and researcher external to the study team extracted data until there was full agreement on the data extracted. All subsequent data extraction was completed by one reviewer (EAB).

### 2.4 Data Analysis

We rated the guideline development manuals, for *each* of our four structured questions, as either ‘Yes’ if PPI guidance was provided for the question or ‘No’ if it was not. We then assessed the reporting of PPI in CPGs as either ‘Yes’ if PPI was reported or ‘No’ if it was not. Finally, we rated the consistency of the PPI reporting in CPGs with the auspicing organisation's guideline development manual. When PPI was reported in the CPG, we rated it as being ‘consistent’ with, ‘somewhat consistent’ with, or ‘contradictory’ to the guidance provided in the guideline development manual). We used ‘Not Applicable’ when no guidance on PPI was provided by the guideline development manual. When a guideline development manual did not provide guidance for a question, but a CPG produced by that auspicing organisation reported details pertinent to the question, we reported this. Criteria for rating the consistency of reporting of PPI in CPGs with the guidance in the auspicing organisation's guideline development manual, with examples is provided in Appendix Table A.2.

## 3 Results

### 3.1 Search Results

**Figure 1:**
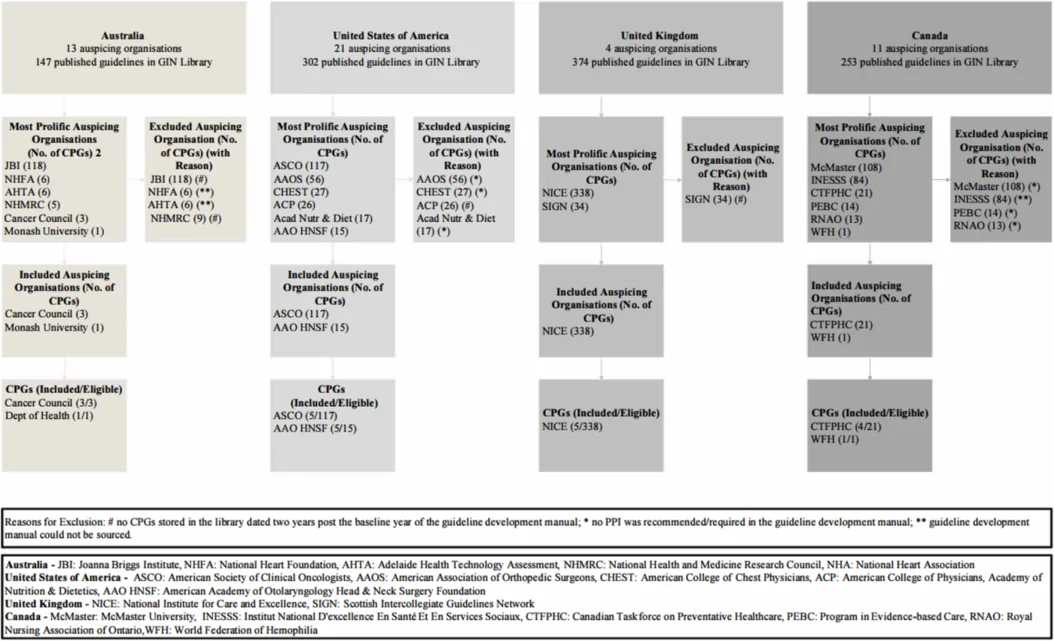
Flow chart showing the process for identifying auspicing organisations and the number and source of CPGs included in this study.

Figure 1 shows the process for identifying auspicing organisations and the number and source of CPGs included in this study.

We identified 49 auspicing organisations in the GIN Library, 13 from Australia, 21 from the US, 4 from the UK, and 11 from Canada. Combined, these organisations auspiced 1,076 guidelines including 147 from Australia, 302 from the US, 374 from the UK and 253 from Canada. Seven auspicing organisations had a guideline development manual/s referencing PP inclusion in CPG development and had produced CPGs that met inclusion criteria. There were two organisations each from Australia (Cancer Council Australia (CCA), and Monash University) [25], the US (American Society of Clinical Oncologists (ASCO), and American Academy of Otolaryngology, Head and Neck Surgery Foundation (AAO HNSF)) [26, 27] and Canada (Canadian Taskforce on Preventive Healthcare (CTFPHC), and World Federation of Hemophilia) [4, 28] and one from the UK (National Institute for Care and Excellence (NICE)) [2, 29]. Of the seven discrete auspicing organisations, the two Australian organisations (CCA, Monash University) both used the same guideline development manual (the 2011 National Health and Medical Research Council (NHMRC) standard for clinical practice guidelines [25]. The UK auspicing organisation, NICE, has a guideline development manual and complementary guide for public involvement in guidelines and standards [2, 29]. These were considered to be one guideline development ‘manual’. As such, there are six guideline development manuals from the seven auspicing organisations.

From these 49 auspicing organisations and 1,076 guidelines, a total of 24 CPGs from seven auspicing organisations were included in this review (Australia = 4, US = 10, UK = 5, Canada = 5).

### 3.2 PPI Guidance in Guideline Development Manuals

Details of PPI guidance provided by the six guideline development manuals of each of the seven auspicing organisations are provided in Appendix Table A.3.

Half (3/6) of the guideline development manuals (AAO HNSF, CTFPHC, NICE) [2, 27, 28, 29] provided guidance on PPI in relation to all four of our PPI reporting questions. All manuals referred to which PPIs to involve and when to involve them in the guideline development process. Most manuals specified that PPI were to be part of a ‘diverse’, ‘multidisciplinary’, ‘expert’ guideline panel, with some manuals specifying minimum representation of PPI. For example, the ASCO guideline development manual specifies that the Expert Panel ‘includes at least one patient representative’ [26]. There was generally limited detail on when to involve PPI, with some manuals indicating PP involvement throughout the guideline development process [2, 26, 27, 29], and or involvement at certain stages or in certain task. For example, the NICE guideline development manual and accompanying guidance specify the PPI are involved in ‘setting the scope of the guideline’, responding to calls for evidence, and commenting on the draft guideline [2, 29]. Four of the six manuals made some reference to how PPI contribute, with guidance ranging from a description of the mode only e.g., ‘face to face meetings’ [4], to roles such as acting as ‘expert witness’ or ‘topic expert’ for a committee [2, 29]. Three of the six manuals did not provide any guidance on from where, and how to recruit PPI [4, 25, 26].

The manuals varied in how prescriptive their language was regarding PPI. Some manuals used directive wording signalling certain conduct was a requirement. For example, the ASCO guidance manual states that ‘All ASCO systematic review‐based guideline products are developed by…’ and ‘includes at least one patient representative’. In contrast, other manuals appear to offer recommendations that set an expectation without requiring adherence, such as the World Federation of Hemophilia manual which states, ‘It is recommended that it should be comprised of a current or former patient’, in relation to who to involve in the guideline development group [4].

### 3.3 PPI Reporting in the CPGs

Ratings of the consistency of reported PPI in CPGs with the auspicing organisation's guideline development manual is shown in Table 1.

**Table 1:** Rating of the consistency of reported PPI in CPGs with the auspicing organisation's guideline development manual.

Auspicing Organisation [Guideline Development Manual]	Clinical Practice Guideline title	Consistency of clinical practice guidelines with guideline development manual guidance on the reporting of:			
		Q1 Who were the patients and publics?	Q2 How and where were the patients and publics recruited?	Q3 At what stage were the patients and publics involved?	Q4 How did the patients and publics contribute their views?
American Society of Clinical Oncology (ASCO)/College of American Pathologists [26]	**Did the guideline development manual provide recommendations/requirements?**	**Yes**	**No**	**Yes**	**Yes**
	Estrogen and Progesterone Receptor Testing in Breast Cancer (2020) [30]	Yes ‐ Consistent	Not Applicable^b^	Yes ‐ Consistent	Yes ‐ Consistent
	Management of Male Breast Cancer (2020) [31]	Yes ‐ Consistent	Not Applicable^b^	Yes ‐ Consistent	Yes ‐ Consistent
	Germline and Somatic Tumor Testing in Epithelial Ovarian Cancer (2020) [32]	Yes ‐ Consistent	Not Applicable^b^	Yes ‐ Consistent	Yes ‐ Consistent
	PARP Inhibitors in the Management of Ovarian Cancer (2020) [33]	Yes ‐ Consistent	Not Applicable^b^	Yes ‐ Consistent	Yes ‐ Consistent
	Optimum imaging strategies for advanced prostate cancer (2020) [34]	Yes ‐ Consistent	Not Applicable^b^	Yes ‐ Consistent	Yes ‐ Consistent
American Academy of Otolaryngology, Head and Neck Surgery Foundation [27]	**Did the guideline development manual provide recommendations/requirements?**	**Yes**	**Yes**	**Yes**	**Yes**
	Clinical Practice Guideline: Sudden Hearing Loss (Update) 2019 [35]	Yes ‐ Consistent	No ‐ Not Reported	Yes ‐ Consistent	Yes ‐ Consistent
	Clinical Practice Guideline: Meniere's Disease 2020 [36]	Yes ‐ Consistent	No ‐ Not Reported	Yes ‐ Consistent	Yes ‐ Consistent
	Clinical Practice Guideline: Otitis Media with Effusion (Update) 2016 [23]	Yes ‐ Consistent	No ‐ Not Reported	Yes ‐ Consistent	Yes ‐ Consistent
	Clinical Practice Guideline: Improving Nasal Form and Function after Rhinoplasty 2017 [37]	Yes ‐ Consistent	No ‐ Not Reported	Yes ‐ Consistent	Yes ‐ Consistent
	Clinical Practice Guideline: Benign Paroxysmal Positional Vertigo (Update) 2017 [38]	Yes ‐ Consistent	No ‐ Not Reported	Yes ‐ Consistent	Yes ‐ Consistent
Cancer Council Australia (CCA) [25]	**Did the guideline development manual provide recommendations/requirements?**	**Yes**	**No**	**Yes**	**No**
	Clinical practice guidelines for keratinocyte cancer: Administrative Report (2019) [39]	Yes ‐ Consistent	Not Applicable^a^, ^b^	Yes ‐ Consistent	Not Applicable^b^
	Clinical practice guidelines for the prevention, early detection and management of colorectal cancer: Administrative Report (2017) [40]	Yes ‐ Consistent	Not Applicable^a^, ^b^	Yes ‐ Consistent	Not Applicable^b^
	Clinical practice guidelines for Surveillance Colonoscopy: Administrative Report (2018) [41]	Yes ‐ Consistent	Not Applicable^a^, ^b^	Yes ‐ Consistent	Not Applicable^b^
Monash University (Australia) [25]	**Did the guideline development manual provide recommendations/requirements?**	**Yes**	**No**	**Yes**	**No**
	Clinical guideline for the diagnosis and management of work‐related mental health conditions in general practice. (2019) [42].	Yes ‐ Consistent	Not Applicable^a^, ^b^	Yes ‐ Consistent	Not Applicable^b^
Canadian Task Force on Preventative Healthcare (CTFPHC) [28]	**Did the guideline development manual provide recommendations/requirements?**	**Yes**	**Yes**	**Yes**	**Yes**
	Guideline on screening for esophageal adenocarcinoma in patients with chronic gastroesophageal reflux disease (2020) [43]	Yes ‐ Consistent	Yes ‐ Somewhat consistent	Yes ‐ Consistent	Yes ‐ Somewhat consistent
	Recommendation on screening adults for asymptomatic thyroid dysfunction in primary care (2019) [44]	Yes ‐ Contradictory	No ‐ Not Reported	No ‐ Not Reported	No‐ Not Reported
	Recommendation on instrument‐based screening for depression during pregnancy and the postpartum period (2022) [45]	Yes ‐ Somewhat consistent	Yes ‐ Somewhat consistent	Yes ‐ Consistent	Yes ‐ Consistent
	Recommendations on screening for primary prevention of fragility fractures (2023) [46]	Yes ‐ Somewhat consistent	Yes ‐ Somewhat consistent	Yes ‐ Consistent	Yes ‐ Consistent
World Federation of Hemophilia [4]	**Did the guideline development manual provide recommendations/requirements?**	**Yes**	**No**	**Yes**	**Yes**
	Guidelines for the Management of Hemophilia, 3rd ed. (2020) [47]	Yes ‐ Consistent	Not Applicable^a^ b	Yes ‐ Consistent	Yes ‐ Consistent
National Institute for Health and Care Excellence (NICE) [2, 29]	**Did the guideline development manual provide recommendations/requirements?**	**Yes**	**Yes**	**Yes**	**Yes**
	Decision‐making and mental capacity (2018) [48]	No ‐ Not reported	No ‐ Not reported	No ‐ Not reported	No ‐ Not reported
	Faltering growth: recognition and management of faltering growth in children (2017). National Institute for Health and Care Excellence (NICE). Faltering growth: recognition and management of faltering growth in children (NG75). London: NICE; 2017.	No ‐ Not reported	No ‐ Not reported	No ‐ Not reported	No ‐ Not reported
	Intrapartum care for women with existing medical conditions or obstetric complications and their babies. (2019) [49]	No ‐ Not reported	No ‐ Not reported	No ‐ Not reported	No ‐ Not reported
	Mental health of adults in contact with the criminal justice system (2017) [50]	No ‐ Not reported	No ‐ Not reported	No ‐ Not reported	No ‐ Not reported
	Workplace health: long‐term sickness absence and capability to work (2019) [51]	No ‐ Not reported	No ‐ Not reported	No ‐ Not reported	No ‐ Not reported

*Note:* Green colour indicates that the guideline development manual provided guidance on this aspect and the guideline authors reported PPI, Red indicates that the guideline development manual provided guidance on this aspect but the guideline authors did not report PPI.

^a^ The manual does not provide guidance, but the CPG provides information on this aspect.

^b^ Not applicable: The guideline development manual did not provide any PPI guidance relevant to the question. As such, the guideline cannot be compared to the manual's guidance.

Nineteen of the 24 CPGs (79%) reported PPI in relation to at least one of the four questions of interest (Appendix Table A.3) including one CPG [44] that reported conduct contrary to the guidance provided in the guideline development manual by not including patients or publics in the guideline development group. Five CPGs did not report information on PPI for any of the four questions [48, 49, 50, 51, 52].

### 3.4 Reported PPI in CPGs According to Guideline Development Manual

Of the nineteen CPGs reporting on PPI in at least one of the four questions of interest, the reporting of PPI was consistent with the recommendations of the guideline development manual in ten CPGs (10/19, 53%). Those ten CPGs [30, 31, 32, 33, 34, 39, 40, 41, 42, 47] were auspiced by ASCO, Cancer Council Australia, Monash University and World Federation of Hemophilia. The five (5/24) CPGs that did not report information on PPI for any of the four questions [48, 49, 50, 51, 52] were all published by an auspicing organisation that provided PPI recommendations for each of the four questions.

Reporting of the PPI questions varied across the 19 CPGs and between CPGs from the same auspicing organisations as follows;

*Q1. Who were the patients and publics involved in developing the CPG?*

All seven guideline development manuals [2, 4, 25, 26, 27, 28, 29] provided guidance relevant to this question and most CPGs (19/24) reported which patients and publics were involved in CPG development. Sixteen CPGs [23, 30, 31, 32, 33, 34, 35, 36, 37, 38, 39, 40, 41, 42, 43, 47] reported PPI consistent with the guidance provided in their auspicing organisation's guideline development manual and two were somewhat consistent [45, 46]. One CPG reported PPI contradictory to the guidance provided [44]. This CPG was a screening CPG, and contrary to the recommendations provided in the guideline development manual, did not include patients or publics in the independent panel of clinicians and methodologists tasked with developing the CPG.

*Q2. From where, and how, were the patients and publics recruited?*

This PPI aspect was reported least often. Four [2, 27, 28, 29] of the seven guideline development manuals provided recommendations for recruiting patients and publics. However, only three of the 14 CPGs [43, 45, 46] published under the auspices of those organisations reported recruitment details, and those details were only somewhat consistent with their auspicing organisations guideline development manuals.

In contrast, five [39, 40, 41, 42, 47] of the ten CPGs published under the auspices of the organisations whose guideline development manuals did not provide guidance on recruitment of patients and publics, did report this information (Appendix Table A.3)

*Q3. At what stage in the CPG development process were the patients and publics involved?*

All seven [2, 4, 25, 26, 27, 28, 29] guideline development manuals provided guidance for which stage PPI was recommended. Eighteen [23, 30, 31, 32, 33, 34, 35, 36, 37, 39, 40, 41, 42, 43, 45, 46, 47, 53] of the 24 CPGs (75%) reported this aspect of PPI, and all 18 CPGs reported PPI consistent with the guidance provided in their auspicing organisation's guideline development manual.

*Q4. How did the patients and publics contribute their views?*

Guidance on how the patients and publics contributed their views during the CPG process was provided by six [2, 4, 26, 27, 28, 29] of the seven guideline development manuals. Of the 19 CPGs auspiced by organisations that provided guidance for this question, 14 (74%) [23, 30, 31, 32, 33, 34, 35, 36, 37, 43, 45, 46, 47, 53] reported this information. Thirteen of those CPGs reported PPI consistently with the guideline development manual's recommendations, and one CPG's reporting of PPI was somewhat consistent [43].

## 4 Discussion and Conclusion

### 4.1 Discussion

This study was a litmus test for the alignment of health organisations' PPI intent and its reporting in their auspiced CPGs. Our results are mixed. Of the 24 CPGs auspiced by organisations that recommended or required PPI in the development of CPGs, 21% (5/24) did not report any PPI in their development. Of the 19 CPGs that did report PPI in their development, only half (10/19) were consistent with the auspicing organisation's guideline development manual in terms of the recommended PPI. The remaining half (9/19) were not consistent or were only somewhat consistent with the PPI guidance. While the manuals of auspicing organisations that provided PPI guidance gave recommendations for most of our four PPI questions, only three of the six guideline development manuals provided guidance on all four questions about who the patients and publics were, how they were recruited, the stage of their involvement, and how their views contributed to the guideline. We consider these four questions basic to an understanding of the PPI process in CPG development.

Strengths of this study include a rigorous, transparent, and reproducible method to assess and analyse the included guidance manuals and CPGs. We used an international CPG repository to systematically identify prolific CPG auspicing organisations, assessed their guideline development manuals for PPI, and then randomly selected CPGs developed under the auspice of these organisations for analysis. However, while the GIN library is one of the world's largest international guideline libraries, it does not include all guidelines produced. Further, the guidelines analysed in this study originated from only four countries and for pragmatic reasons, we only included a small sample of CPGs. Of the 1,076 CPGs originating from our four countries of interest at the time of data collection, we included 24 CPGs. By limiting our sample to guidelines included in the GIN library and to guidelines produced in selected countries (Australia, Canada, UK, and US), the findings may have limited generalisability to guidelines from other developed and developing countries. Further, our findings reflect the guideline manual recommendations for PPI at the time of our search, and organisational guidance may have since changed. Although only one study author extracted data from the guideline development manuals and included CPGs, methods were implemented to minimise errors arising from this approach. In addition, though a protocol for this research study was written prior to the start of the study, the protocol is not publicly available.

The guideline development manuals we evaluated often did not provide guidance on two PPI questions: How and where patients and publics were recruited? and How patients and publics contributed their views? Where the patients and publics were recruited from is important to understand because the aim is to recruit appropriate people with different skills and experience who can represent the guideline target audience without focusing on their individual experience or agenda [54]. Guideline developers and patient organisations have identified lack of interest from patient and public members to get involved in guideline development as a barrier to achieving sufficient representation on guideline groups so understanding from where and how to recruit patients and public would be expected to assist [55]. Similarly, understanding how patients' and publics' views contributed to the development of the CPG would allow blending of patients' and publics' preferences with scientific evidence to develop recommendations ensuring that PPI is genuine [56].

There appear to be improvements in the reporting of PPI. A decade ago, the NHMRC [1] reported that of the 1,046 CPGs auspiced by the NHMRC and published between 2005 and 2013 for use in clinical practice in Australia only 14% reported PPI, 46% did not report PPI and the information provided in the remaining 40% of guidelines weas insufficient to determine whether PPI occurred or not. That was despite the 1999 and 2011 guides to the development of CPGs recommending PPI [25, 57] and more than 25% of the published CPGs referencing NHMRC documents in their development process. Happily, and in contrast, we found that the four randomly selected CPGs (dating between 2017 and 2019) published under auspicing organisations referencing the NHMRC guideline development manual all reported PPI complying with the manual's guidance.

The consistency of reported PPI in CPGs with the auspicing organisations' recommendations seen in our study is comparable to that seen in other studies. A German study [16] reported a descriptive analysis of 270 CPGs, limited to only those developed by member societies of one auspicing organisation, the Association of Scientific Medical Societies in Germany (AWMF). The AWMF follows the recommendations of German Guideline Assessment Tool (DELBI) which requires patients to be involved in the development of CPGs, ideally through membership of the author group. Assessed against the DELBI, evidence of PPI in CPG development was reported in 233/270 CPGs (86%). Full consistency with the recommendations, specifically the inclusion of patients in the guideline authorship group and the provision of voting rights, was reported in 148/270 CPGs (55%) which is broadly consistent with the current finding of 53%. In a recent update to this study however, the inclusion of patients in the guideline authorship group and the provision of voting rights had risen to 73% [20]. An analysis of 76 CPGs by US authors [14], reported 61% of those CPGs fully complied with the auspicing organisation's guideline development manual. However, the study was published as a conference abstract only (with no response to our email correspondence requesting information) so the study method was not available.

Our study raises the following question: is the lack of consistency between CPGs and the recommendations of the auspicing organisation's guideline development manual a reporting or a compliance issue, or both? Without a standard approach to reporting PPI in CPGs it remains difficult to answer. Well‐intentioned, genuine PPI must be clear in language and process as well as transparent and repeatable. Otherwise, PPI in CPG development remains an expectation rather than routine practice. For that reason, guideline development manuals should be written with the intention of providing a process that is transparent and through complete reporting of that process, is repeatable.

Where does the responsibility for the commitment to that intent lie? Is it with the guideline authors to comply with and report the PPI process recommended by the auspicing organisation under which the CPG is published? Or is it with the organisation that published the guideline development manual and auspices the CPGs, to monitor commitment to intent for PPI processes? Barriers to reporting suggested by guideline authors included a lack of a framework for reporting and uncertainty about where in the published CPG to include PPI information [56]. Encouragingly one of the surveyed guideline authors recommended mandatory reporting of the ‘when’ and ‘how’ of the PPI process to promote the development of CPGs informed by patient preferences. A recent descriptive report on consumer engagement in development of living guidelines [58] captured the experiences of guideline developers and consumers, defined as patients, carers, the public and their representatives, but did not address the reporting of processes. It is noteworthy that despite GIN's standards for CPG development and the associated reporting checklist for voluntary submission along with the CPG, none of the guideline authors submitted the reporting checklist. Further research is required to capture the opinions of CPG auspicing organisations and CPG guideline authors to answer these questions to improve compliance and/or reporting PPI in CPG development. Further understanding of the factors that may facilitate or imped the reporting of PPI in guideline development including examination of elements such as whether manuals use language that signals a requirement or a suggestion, the availability of reporting templates and the influence of editorial policies will strengthen both the practice and reporting of PPI.

Our review of guidelines and manuals noted inconsistent terminology used when describing PPI in CPG development. Descriptions of the patients and publics involved included ‘patient representative’ (ASCO), ‘consumers’(NHMRC) and ‘lay members’ (NICE). More helpfully, recruited patients and publics for a screening CPG were described as ‘English‐speaking members of the public who are targets of the CPG but who do not have the disease’ (CTFPHC). Inconsistent terminology, particularly in the absence of definitions and reporting guidance, confuses perceptions of which patients and publics have been and should be involved in developing CPGs and hinders establishment of a model of best practice for PPI in CPG development. A recently developed checklist, GIN‐McMaster Guideline Development Checklist extension for engagement, subsumes patients and publics into the term ‘interest‐holders’ but does offer useful definitions of ‘patients’ and ‘public’ [59].

### 4.2 Conclusion

Although there are improvements in reporting PPI in the development of CPGs, it remains inconsistent. To improve PPI reporting in CPGs we require auspicing organisations and their guideline authors/developers to (i) encourage PPI in CPG development; (ii) report PPI in the CPG development process and (iii) for CPG developers to adhere to the PPI process and auspicing organisations to advocate for compliance.

Our aim is not to be prescriptive about auspicing organisations' PPI in CPG development methods, but we strongly recommend consistent reporting so that the PPI process in CPG development can be appraised to establish best practice. A consensus‐developed reporting checklist specifically for PPI in the development of CPG development would facilitate this for guideline authors.

### 4.3 Practice Implications and Future Research

Reporting of PPI in CPGs appears arbitrary. Future research should aim to understand what would facilitate uptake and motivate appropriate reporting of PPI in guideline development by guideline authors/developers. For well‐intentioned, genuine PPI in CPG development to be acknowledged and replicated, the language should be consistent (or at least defined) and the process should be clearly reported. Otherwise, it will remain an intent instead of best practice.

## Author Contributions

**Elizabeth Ann Bryant:** Conceptualization, investigation, writing – original draft, methodology, validation, visualization, writing – review and editing, software, project administration, formal analysis, data curation, resources. **Sharon Sanders:** Conceptualization, investigation, methodology, validation, visualization, resources validation, supervision, writing – review and editing. **Rae Thomas:** Conceptualization, investigation, methodology, visualization, validation, supervision, writing – review and editing, resources, project administration.

## Funding

The authors received no specific funding for this work.

## Conflicts of Interest

The authors declare no conflicts of interest.

## Supporting information

Supplementary Material.

## Data Availability

All data relevant to the study are included in the article or uploaded as supplementary information.
