# Supplementary material for: Patient and Public Involvement Reporting Consistency: Analysis of 24 Clinical Practice Guidelines From 4 Countries
Source: Clin Public Health Guidel. 2026 Feb 4;3(2):e70061. doi: 10.1002/gin2.70061 (PMC13563320; doi:10.1002/gin2.70061)
Supplement: Supplementary file 1 [file GIN2-3-e70061-s001.docx]

**Patient and Public Involvement Reporting Consistency: Analysis of 24 Clinical Practice Guidelines from 4 countries.**

Supplementary Material

Appendix

Table A.1 Terminology used in this study, with an example

| **Phrase** | **Definition** |
| --- | --- |
| Auspicing organisation | The organisation supporting the development and publishing of the CPG, and the organisation responsible for the CPG development manual. For the CPG titled *Clinical Practice Guideline: Otitis Media with Effusion (Update), 2016* [20] the auspicing organisation is the American Academy of Otolaryngology, Head and Neck Surgery Foundation. |
| Guideline development manual | The manual from the auspicing organisation that provides guidance on how to develop a CPG. For the American Academy of Otolaryngology, Head and Neck Surgery Foundation the guidance manual is *Clinical practice guideline development manual: a quality-driven approach for translating evidence into action, 2013* [26] |
| Clinical practice guideline (CPG) | The CPG published by the Guideline authors that provides recommendations on how to diagnose and/or treat a medical condition and intended to facilitate informed decision making and optimise patient care [10]. For example, *Clinical Practice Guideline: Otitis Media with Effusion (Update), 2016* [20] is a CPG |
| Guideline authors | The authorship team who wrote the published CPG (e.g., RM Rosenfeld, JJ Shin, et al are the guideline authors of the CPG titled *Clinical Practice Guideline: Otitis Media with Effusion (Update), 2016* [20] |
| Study authors | The authors of the current study – *Patient and Public Involvement Reporting Consistency: Analysis of 24 Clinical Practice Guidelines across 4 countries.* |

Appendix

Table A.2 Criteria for rating the consistency of reporting of PPI in CPGs with the guidance provided, with example Data extracted from the guideline development manuals and CPGs

|  | Example | |
| --- | --- | --- |
| Rating of PPI reporting in the guideline | Recommendation/requirement in the guideline development manual | Example quote from CPG and reason for rating |
| **Yes – Consistent**  When PPI was reported and was consistent with the recommendations/requirements of the guideline development manual. | Example requirement for Question 1: Who are the patients and publics involved in developing the guideline?  *“Once a topic is approved by the CPGC (Clinical Guideline Practice Committee) an Expert Panel is assembled. All ASCO systematic review-based guideline products are developed by a multidisciplinary Expert Panel…. Each expert panel should have a representative from the ASCO Practice Guidelines Implementation Network (PGIN) and at least one patient representative.”*  Quote from: The American Society of Clinical Oncology (ASCO) Guideline Methodology Manual, 2019, p.4. [1] | Example CPG reporting for Question 1: Who are the patients and publics involved in developing the guideline?  *“Expert Panel comprising 30 members from all the partner organizations (herein, the*  *Expert Panel), which included a patient representative - Westley Scholes, a PGIN representative plus a staff/health research methodologist. Westley Scholes is a prostate cancer survivor.”*  Quote from: Optimum imaging strategies for advanced prostate cancer: ASCO Guideline, 2020, p.1968. [6] |
|  | Examples | |
| **Yes – Somewhat Consistent**  When PPI was reported and was somewhat consistent with some but not all the guideline development manual recommendations/requirements. | Example requirement for Question 4: How does PPI  contribute?  *“Phase 1. RAND Appropriateness Method (RAM) Phase 2. RAND Appropriateness Method (RAM) Phase 3. The SMH KT Program uses focus groups or interviews to* | Example CPG reporting for Question 4: How does PPI  contribute?  *Patient outcomes assessed using the GRADE method, not the RAM.* |

|  | *obtain feedback on the content, format, aesthetics and usefulness of patient KT tools developed to accompany the CTFPHC guideline”.*  Quote from: Canadian Taskforce on Preventive Healthcare (CTFPHC) Patient Engagement Protocol, 2017, pp. 5-13 [18] | Quote from: Guideline on screening for esophageal carcinoma in patients with chronic gastroesophageal reflux disease 2020, p.E773 [19] |
| --- | --- | --- |
|  | Examples | |
| **Yes - Contradictory**  When PPI was reported and was contradictory to the guideline development manual recommendations/requirements | Example requirement for Question 1: Who are the patients and publics involved in developing the guideline?  *“Patient engagement conducted through the Knowledge Translation group at St. Michael’s Hospital, Toronto.*  *Phase 1. Approx. 16-20 English-speaking members of the Canadian public - not practicing health professionals; but are intended targets of the guideline and partners or caregivers of intended targets. Phase 2.*  *Same as for Phase 1. Phase 3. Approx. 6-8 English-speaking Canadians for whom the guideline and patient KT tools will be relevant”*  *For all guideline topics, individuals who already have the disease and/or have conflicts of interest relevant to the guideline (e.g.., membership in a relevant disease-specific organisation or owning shares in a disease- relevant company) are excluded.”* | Example CPG reporting for Question 1: Who are the patients and publics involved in developing the guideline?  “The task force is an independent panel of clinicians and methodologists that makes recommendations on primary and secondary prevention in primary care”  Note: A ‘working group’ was mentioned but membership was not defined.  Quote from: Recommendation on screening adults for asymptomatic thyroid dysfunction in primary care, 2019, p.E1275. [20] |

|  | Quote from: Canadian Taskforce on Preventive Healthcare (CTFPHC) Patient Engagement Protocol, 2017, p.6,9,12 [18] |  |
| --- | --- | --- |
| **Not Applicable**  The guideline development manual did not provide any PPI recommendation/requirement relevant to the question. Therefore, any or lack of information provided in the guideline cannot be compared to the manual’s recommendation/requirement. NA will be reported but any quotes will be noted. | Example requirement for Question 2: How to recruit patients and publics and from where?  *No guidance provided.*  Observation from: Procedures and requirements for meeting the 2011 NHMRC standard for clinical practice guidelines. [13] | Example CPG reporting for Question 2: How to recruit patients and publics and from where?  *“Recruited from the Consumer CAN Network, an advocacy group active in the areas of cancer diagnosis, information, treatment, research, support, care, survivorship, and policy, that works with decision-makers to hear the patient perspective.”*  Quote from: Clinical practice guidelines for the prevention, early detection and management of colorectal cancer: Administrative Report, 2017, p.13. [15] |
|  | Examples | |
| **No – Not Reported**  The guideline development manual provided a PPI recommendation/requirement relevant to the question, but the guideline did not report the information. | Example requirement for Question 1. Who are the patients and publics involved in developing the guideline?  *“All our Committees include at least 2 lay members (people with personal experience of using health or care services, or from a community affected by the guideline.”*  Quote from: Developing NICE guidelines: the manual, 2014, p.5 [25] | Example CPG reporting for Question 1. Who are the patients and publics involved in developing the guideline?  *GIN Library has an abbreviated form of the guideline which notes at the end of the document that more information can be found by following the hyperlinks to the full guideline and how the guideline was developed. Search details were described but both the abbreviated and full guideline note:* |

|  | *“For the purposes of this guide the term ‘lay member’ is used to describe the*  *patients, carers, service users, experts by experience, survivors, and members of*  *the public who contribute to the development of NICE guidance and quality*  *standards by sitting on a NICE committee.*  *This includes people with a relevant*  *care or support need, condition or disability, family and friends who provide*  *unpaid care for them, people who work at voluntary and community sector organisations and people who have an advocacy role.”*  Quote from: NICE's approach to public involvement in guidance and standards: a practical guide, 2015,  p.5 [26] | *"This guideline was developed in accordance with the process and methods set out in Developing NICE guidelines: the manual."*  Quote from: Decision-making and mental capacity (NG108), 2018, Appendix A. [27] |
| --- | --- | --- |

Appendix

Table A.3 Data extracted from the guideline development manuals and CPGs

UNITED STATES

# American Society of Clinical Oncology

|  | Q1 Who to involve? | Q2 How to recruit and  from where? | Q3 When to involve? | Q4 How does PPI  contribute? | * G-I-N Standards for Clinical Practice Guidelines. |
| --- | --- | --- | --- | --- | --- |
| ASCO Guidelines Methodology Manual [1] | Once a topic is approved by the CPGC an Expert Panel is assembled. All ASCO systematic review-based guideline products are developed by a multidisciplinary Expert Panel, of approx 15 members, which includes at least one patient representative and is supported by ASCO guidelines staff with health research methodology expertise. | No guidance provided. | The Expert Panel is involved throughout the development process.  The patient representative assists in advising on patient communication during the initial drafting. Patients/patient advocacy groups are engaged for open comment on the draft guideline. This is in the final review process to identify errors/gaps. | Email, face-to-face meetings. |  |
| Estrogen and Progesterone Receptor Testing in Breast Cancer: ASCO/College of American Pathologists Guideline Update [2] | Yes, page 1349.  Patient representative (n= 1/20) on the expert panel is a cancer survivor | N/A | Yes, page 1352, 1361.  Expert panel involved throughout; however, author contributions at the end of the published article mention the | Yes, page 1352, 1361.  Face-to-face meetings, email | No |

|  |  |  | patient representative by name in data analysis and interpretation stage. All authors noted as contributing to manuscript writing and final approval of manuscript. Open comment period confirmed. |  |  |
| --- | --- | --- | --- | --- | --- |
| Management of Male Breast Cancer: ASCO Guideline [3] | Yes, page 1865.  Patient representative (n=1/13) on the expert panel. PhD student | N/A | Yes, page 1852, 1861.  Expert Panel contributed to the development of the guideline, critical review, and final guideline recommendations (including open comments). Author contributions at the end of the article confirm that all authors contributed to data analysis and interpretation, manuscript writing, final approval of manuscript and were accountable for aspects of the work. Open comment period confirmed. | Yes, page 1852.  The Expert Panel met in person, via teleconference and corresponded through email. | No |

| Germline and Somatic Tumor in Epithelial Ovarian Cancer: ASCO Guideline [4] | Yes, page 1223.  Patient representative (n=1/13) on the expert panel is an ovarian cancer survivor. | N/A | Yes, page 1224, 1242.  Expert Panel contributed to the development of the guideline, critical review, and final guideline recommendations (including open comments). Author contributions at the end of the article confirm that all authors contributed to data analysis and interpretation, manuscript writing, final approval of manuscript and were accountable for aspects of the work. Open comment period confirmed. | Yes, page 1223.  The Expert Panel met in person, via teleconference and corresponded through email | No |
| --- | --- | --- | --- | --- | --- |
| PARP Inhibitors in the Management of Ovarian Cancer: ASCO Guideline [5] | Yes, page 3471.  Patient representatives (n=2/17) on the expert panel are patient advocates from ovarian cancer patient groups. | N/A | Yes, page 3471-3472.  Expert Panel contributed to the development of the guideline, critical review, and final guideline recommendations (including open comments). Author contributions at the end of the article confirm that all authors contributed to | Yes, page 3471.  The Expert Panel met in person, via teleconference and/or webinar and corresponded through email. | No |

|  |  |  | conception and design, data analysis and interpretation, manuscript writing, final approval of manuscript and were accountable for aspects of the work. Open comment period confirmed. |  |  |
| --- | --- | --- | --- | --- | --- |
| Optimum imaging strategies for advanced prostate cancer: ASCO Guideline [6] | Yes, page 1968.  Patient representative (n=1/30) on the expert panel is a PGIN (Practice Guideline International Network) representative and a prostate cancer survivor. | N/A | Yes, page 1969, 1991,  1993.  Expert Panel contributed to the development of the guideline, critical review, and final guideline recommendations (including open comments). Author contributions at the end of the article confirm that all authors contributed to conception and design, collection and assembly of data, data analysis and interpretation, manuscript writing, final approval of manuscript and were accountable for aspects of | Yes, page 1969, 1991.  The Expert Panel met in person several times as well as via teleconference and/or webinar and corresponded through e- mail. | No |

|  |  |  | the work. Open comment period confirmed. |  |  |
| --- | --- | --- | --- | --- | --- |

UNITED STATES

# AAO HNSF

|  | Q1 Who to involve? | Q2 How to recruit and from where? | Q3 When to involve? | Q4 How does PPI contribute? | * G-I-N Standards for Clinical Practice  Guidelines. |
| --- | --- | --- | --- | --- | --- |
| Clinical practice  guideline development  manual: a quality-  driven approach for  translating evidence  into action. [7] | The GDG – 12 to 18  members including 2  consumer  representatives. A  ’consumer’ broadly  defined as a recipient of  health care (in contrast to  a provider) who provides  a layperson perspective  for the group.  Considered “experts” in  advocating for the  public, patients, and their  proxies. | Identified by  organizational  leadership, partner  organizations and staff  liaisons. | Throughout the  guideline development  process including a 2-  week period of public  comment. | Specific assignments  and deadlines assigned  during conference  calls, three in total.  Two in-person  meetings. Public  review of the guideline  with comments posted  online. The final  guideline is distributed  electronically, and  members respond.  Consumer advocacy  groups are recruited to  review the original  guideline, prepare an  executive summary. |  |

| Clinical Practice Guideline: Sudden Hearing Loss (Update) [8] | Yes, page S6,  the Guideline Update Group (GUG) included ‘experts in consumer advocacy’ but no confirmation of how many.  The previous CPG was reviewed by a panel, with no PPI, to determine if Key Action Statements (KAS) needed amendment. | Not Reported | Yes, page S6-7, S9,  the GUG classified the role of patient preferences  Details included in how the PPI manifested. | Yes, page S6-7,  Three conference calls, one in-person meeting to define the scope and objectives of updating the guideline, reviewed comments from the expert panel review for each KAS, identified other quality improvement opportunities and reviewed the literature search results. | No |
| --- | --- | --- | --- | --- | --- |
| Clinical Practice Guideline: Meniere’s Disease [9] | Yes, page S6,  the Guideline Development Group (GDG) consisted of 21 panel members including ‘experts in consumer advocacy’ but no confirmation of how many. Confirmed that the method for development was as per the guideline manual. | Not Reported | Yes, page S6  Details included in how the PPI manifested. | Yes, page S6,  in a series of conference calls the GDG defined the scope and objectives of the proposed guideline; two in-person meetings during the 18-month development period; internal electronic review and feedback on each guideline draft, multidisciplinary peer review, open public comment. | No |

| Clinical Practice Guideline: Otitis Media with Effusion (Update) [10] | Yes, page S2, S6  noting that there was change in methodology for this update to include consumer advocates to the Guideline Update Group (GUG)  The previous CPG was reviewed by a panel, with no PPI, to determine if Key Action Statements (KAS) needed amendment.  Confirmed that the method for development was as per the guideline manual. | Not Reported | Yes, page S6  Details included in how the PPI manifested. | Yes, page S6,  the GUG had several conference calls and one in-person meeting during which it defined the scope and objectives of updating the guideline, reviewed comments from the expert panel review for each key action statement, identified other quality improvement opportunities, and reviewed the literature search results; reviewed each guideline draft, multidisciplinary peer review, open public comment. | No |
| --- | --- | --- | --- | --- | --- |
| Clinical Practice Guideline: Improving Nasal Form and Function after Rhinoplasty [11] | Yes, page S6,  the Guideline Development Group (GDG) consisted of 16 panel members including ‘experts in consumer advocacy’ but no | Not Reported | Yes, page S7  Details included in how the PPI manifested. | Yes, page S7,  In a series of conference calls, the working group defined the scope and objectives of the proposed guideline.  During the 16 | No |

|  | confirmation of how many.  Confirmed that the method for development was as per the guideline manual. |  |  | months development, the group met twice; internal electronic review and feedback on each guideline draft; modified an advanced draft of the guideline; the final guideline draft underwent extensive external peer review; comments were compiled and reviewed by the panel’s chair and co-chairs, and a modified version of the guideline was distributed and approved by the guideline development  panel |  |
| --- | --- | --- | --- | --- | --- |
| Clinical Practice Guideline: Benign Paroxysmal Positional Vertigo (Update) [12] | Yes, page S2, S6  noting that there was change in methodology for this update to include a patient advocate to the Guideline Update Group (GUG)  The previous CPG was reviewed by a panel, with no PPI, to determine if Key Action | Not Reported | Yes, page S6  Details included in how the PPI manifested. | Yes, page S6  The guideline update group had several conference calls and 1 in-person meeting, during which it defined the scope and objectives of updating the guideline, reviewed comments from the expert panel review | No |

|  | Statements (KAS) needed amendment. Guideline Update Group (GUG) included ‘experts in consumer advocacy’ but no confirmation of how many.  NB – terminology change withing the guideline.  Confirmed that the method for development was as per the guideline manual. |  |  | for each key action statement, identified other quality improvement; reviewed each guideline draft, multidisciplinary peer review, open public comment. opportunities, and reviewed the literature search results. |  |
| --- | --- | --- | --- | --- | --- |

AUSTRALIA

# Cancer Council Australia

|  | Q1 Who to involve? | Q2 How to recruit and  from where? | Q3 When to involve? | Q4 How does PPI  contribute? | * G-I-N Standards for Clinical Practice Guidelines. |
| --- | --- | --- | --- | --- | --- |
| Procedures and | Consumers participate  in the guideline  development, and the  processes employed to  recruit, involve and  support consumer  participants are  described. Public  consultation for the draft  guideline. The guideline  is required to consult | No guidance provided | Public consultation is  involved with 30 days  for submissions to be  submitted. | No guidance provided |  |
| requirements for |  | for recruiting |  |  |  |
| meeting the 2011 |  | consumer |  |  |  |
| NHMRC standard for |  | representatives. |  |  |  |
| clinical practice |  | Recruit public |  |  |  |
| guidelines [13] |  | consultation through |  |  |  |
|  |  | one major national |  |  |  |
|  |  | daily newspaper and |  |  |  |
|  |  | summary of the draft |  |  |  |
|  |  | guideline (or a link to |  |  |  |
|  |  |  |  |  |  |

|  | with interested organisations (referred to as stakeholders). | it) is provided in that national newspaper. |  |  |  |
| --- | --- | --- | --- | --- | --- |
| Clinical practice guidelines for keratinocyte cancer: Administrative Report [14] | Yes, page 6-7.  The Keratinocyte Cancer Guidelines Revision Working Party consisted of two consumer representatives.  Additional public consultation. The two chosen are Ann Strokon and Danny Brennan. No available information on these individuals, including disclosure of interests. | Record N/A but information provided, page 11.  Both consumer representatives were engaged through recommendations from Working Party members. | Yes, page 13-15.  The consumer representatives were involved in reviewing the draft guideline content developed by section leads and subcommittee and attended face-to-face working party meetings pre and post public consultation to approve guideline content.  Public consultation for 30 days with key organisations that were notified of public consultation. | Record N/A but information provided, page 11, 13.  Mentioned that "The consumer representatives are invited to review content and attend meetings of the Working Party and are involved in the development of the guidelines content” but did not specify their involvement in guideline development. Working Parties were used to develop clinical questions and determine the scope of the content and later guidance revisions. | No |

| Clinical practice guidelines for the prevention, early detection and management of colorectal cancer: Administrative Report [15] | Yes, page 4.  Two members of the Working Party were consumer advocates (Jeff Cuff and Jillian Arnott). Jillian Arnott is a long-term survivor and support group participant. Jeff Cuff is the husband of a patient who passed away, and the founder of the Shirley Cuff Colon Cancer Research Fund. | Record N/A but information was provided, page 13.  Recruited from the Consumer CAN Network, an advocacy group active in the areas of cancer diagnosis, information, treatment, research, support, care, survivorship and policy, that works with decision-makers to hear the patient perspective. | Yes, page 15-17.  Working Party worked with the Cancer Council Australia systematic review team to draft clinical questions in line with specialty.  Reviewed the draft and shared ideas during the meeting, came to consensus.  The Working Party members reviewed the draft guidelines content developed by the section leads and attend the  face-to-face Working Party meetings pre- and post- public consultation to approve the guidelines contents.  Public consultation for 30 days and consumer groups and decision- makers were notified of its existence. | Record N/A but information provided, page 14-15.  Emails, teleconferences and face-to-face meetings. | No |
| --- | --- | --- | --- | --- | --- |
| Clinical practice guidelines for Surveillance Colonoscopy: | Yes, page 5-6.  Two members of the Working Party were | Record N/A but information was provided, page 9. | Yes, page 5, 10, 14.  The Working Party members reviewed the | Record N/A but information provided, page 12. | No |

| Administrative Report [16] | consumer advocates (Jeff Cuff and Jillian Arnott). Jillian Arnott is a long-term survivor and support group participant. Jeff Cuff is the husband of a patient who passed away, and the founder of the Shirley Cuff Colon Cancer Research Fund. | Recruited from the Consumer CAN Network, an advocacy group active in the areas of cancer diagnosis, information, treatment, research, support, care, survivorship and policy, that works with decision-makers to hear the patient perspective. | draft guidelines content developed by the section leads and attend the  face-to-face Working Party meetings pre- and post- public consultation to approve the guidelines content.  Public consultation for 30 days and consumer groups and decision- makers were notified of its existence. | Emails, teleconferences and face-to-face meetings. |  |
| --- | --- | --- | --- | --- | --- |

AUSTRALIA

# Monash University

|  | Q1 Who to involve? | Q2 How to recruit and  from where? | Q3 When to involve? | Q4 How does PPI  contribute? | * G-I-N Standards for Clinical Practice Guidelines. |
| --- | --- | --- | --- | --- | --- |
| Procedures and requirements for meeting the 2011 NHMRC standard for clinical practice guidelines [13] | Consumers participate in the guideline development, and the processes employed to recruit, involve and support consumer participants are described. Public consultation for the draft guideline. The guideline is required to consult with interested | No guidance provided for recruiting consumer representatives.  Recruit public consultation through one major national daily newspaper and summary of the draft guideline (or a link to it) is provided in that national newspaper. | Public consultation is involved with 30 days for submissions to be submitted. | No guidance provided |  |

|  | organisations (referred to as stakeholders). |  |  |  |  |
| --- | --- | --- | --- | --- | --- |
| Clinical guideline for the diagnosis and management of work- related mental health conditions in general practice. [17] | Yes, page 28  there was a representative from Mental Health Australia – a consumer with a lived experience of a work-related mental health condition GDG | Record N/A  and note that no information was provided re recruitment of the consumer rep.  Record N/A  but note that the CPG and supporting documentation was sent to 39 individuals and 101 organisations nationally; website announcements by Monash University and NHMRC; steering committee contacts | Yes, page 33  Public consultation for 60 days | Record N/A  and note that information was provided page 28  The GDG convened 8 times over the course of CPG development – 5/8 face-to-face and 3/8 teleconference. | No |

CANADA

# Canadian Task Force on Preventative Healthcare

|  | Q1 Who to involve? | Q2 How to recruit and  from where? | Q3 When to involve? | Q4 How does PPI  contribute? | * G-I-N Standards for Clinical Practice Guidelines. |
| --- | --- | --- | --- | --- | --- |
| Canadian Task Force on  Preventative Healthcare  Patient Engagement  Protocol [18] | Patient engagement  conducted through the  Knowledge Translation  group at St. Michael’s  Hospital, Toronto.  1. Approx. 16-20  English-speaking  members of the  Canadian public - not  practicing health  professionals; but are  intended targets of the  guideline and partners  or caregivers of  intended targets. 2.  Same as for Phase 1. 3.  Approx. 6-8 English-  speaking Canadians for  whom the guideline and  patient KT tools will be  relevant | 1. To recruit a diverse  sample recruitment, ads  are posted on the  CTFPHC website and  on public advertisement  websites (Craigslist).  Emailing directly  members of the public  known to SMH KT and  have expressed interest  in contributing  feedback. Applicants  complete an online  survey and are selected  based on the  demographic, health  and health equity  inclusion criteria  specific to the  guideline. Applicants  who already have the  disease are excluded. 2.  Same as for Phase 1 3.  Same as for Phases 1  and 2. | 1. Rate the screening  outcomes relevant to a  guideline topic  that are most important to  consider during decision  making. 2. Participants  provide their perspectives  on the screening  outcomes for a particular  guideline topic. In this  case, however,  participants also receive  information from the  systematic review about  the relative likelihood of  each  outcome.3. Participants  are engaged in usability  testing of patient KT  tools. CTFPHS  incorporated a contextual  question on patient  preferences in all  evidence reviews, a  process that involves a  literature search on patient preferences and values specific to the analytic framework of each guideline. | Phase 1. RAND  Appropriateness  Method (RAM) to rate screening outcomes most important to consider during decision making Phase 2.RAND Appropriateness  Method (RAM) to provide perspectives on the outcomes with information from the systematic review.3. The  SMH KT Program uses  focus groups or  interviews to obtain  feedback on the  content, format,  aesthetics and  usefulness of patient  KT tools developed to  accompany the  CTFPHC guideline. |  |

| Guideline on screening for esophageal adenocarcinoma in patients with chronic gastroesophageal reflux disease [19] | Yes, page E773.  Guideline was developed using a 5 Member Panel consisting of some of the authors. Phase 2 involved patients, including 17 men and women with GERD. | Yes, Partial, page E773.  Recruited 17 men and women with gastroesophageal reflux disease (GERD). No further detail provided. | Yes, page E773. | Yes, Partial, page E773.  Patient outcomes assessed using the GRADE method, not the RAM. | No |
| --- | --- | --- | --- | --- | --- |
| Recommendation on screening adults for asymptomatic thyroid dysfunction in primary care [20] | No, page E1275  “The task force is an independent panel of clinicians and methodologists that makes recommendations on primary and secondary prevention in primary care” | Not reported | Not reported | Not reported | No |
|  | A ‘working group’ was mentioned but membership was not defined. |  |  |  |  |
| Recommendation on instrument-based | Yes - partial page E985, | Yes - partial, page E985, | Yes, page E985, | Yes, page E985, | No |

| screening for depression  during pregnancy and  the postpartum period  [21] | confirmed patient  engagement activities.  Phase 1, 15 female  pregnant & postpartum  participants.  Phase 2, 14 different  female pregnant &  postpartum participants.  Not confirmed that the  participants did not  have a current diagnosis  of depression. | Recruited via public ads  on Craigslist and Kijiji,  no further details  provided. | Full description of PPI  activities with  confirmation that the  outcomes rated as critical  or important were  considered during  guideline development.  Provided feedback on  key messages and  opinions on interactive  decision aids | Surveys, focus groups |  |
| --- | --- | --- | --- | --- | --- |
| Recommendations on screening for primary prevention of fragility fractures [22] | Yes, Partial, Appendix 6 online version.  Participants were English-speaking men and women in Canada who are either members of the screening population, or those who have experienced a fragility fracture and/or been previously or currently diagnosed with osteoporosis or osteopenia.  **Note:** including participants who have experienced a fragility fracture and/or | Yes, Partial, Appendix 6 online version.  We recruited participants by posting recruitment advertisements on public advertisement websites (i.e., Craigslist and Kijiji). We also circulated recruitment advertisements in the newsletters and internal communications of relevant organizations: Osteoporosis Canada, Bone and Joint Health in Alberta, Strategy for Patient Oriented Research Evidence | Yes, page E644  Full description of PPI activities with confirmation that the outcomes rated as critical or important were considered during guideline development. Provided feedback on key messages and opinions on interactive decision aids | Yes, page E642, Surveys, focus groups | No |

|  | osteoporosis goes against Recruitment Recommendations | Alliance, and the Canadian Frailty Network.  **Note:** including participants who have experienced a fragility fracture and/or osteoporosis goes against Recruitment Recommendations |  |  |  |
| --- | --- | --- | --- | --- | --- |

CANADA

# World Federation of Hemophilia

|  | Q1 Who to involve? | Q2 How to recruit and  from where? | Q3 When to involve? | Q4 How does PPI  contribute? | * G-I-N Standards for Clinical Practice Guidelines. |
| --- | --- | --- | --- | --- | --- |
| Clinical Practice Guidelines We Can Trust (IOM,  2011) [23] | "Multidisciplinary and balanced". It is recommended that it should be comprised of a current or former patient, and a patient advocate or patient/consumer organisation in the development group. | No guidance provided. | Involved in the guideline development group at least at the time of clinical question formulation and draft CPG review. External reviewers should comprise a full spectrum of relevant stakeholders, including patients, and representatives of the public. A draft of the CPG at the external review stage or immediately following it (i.e., prior to the final draft) should be made available to the general public for comment. | Face-to-face meetings, formal consensus methods. Strategies to increase effective participation of patient and consumer representatives, including training in appraisal of evidence, should be adopted by GDGs. |  |
| Guidelines for the management of hemophilia, 3^rd^ ed [24] | Yes, page 149. | Record N/A but information was provided, page 149. | Yes, page 149. | Yes, page 149. | No |

|  | The Guidelines Process Task Force was comprised on patients, hematologists that were not involved in the process of guideline development. Patients and caregivers comprised 25% of the panel. | Not stated specifically, but "panellists were recruited from diverse demographic, geographic, and socioeconomic contexts to ensure the global relevance of these guidelines". | All panellists involved in topic selection, generating evidence, consensus process (modified Delphi) and guideline drafting.  Further involved in the guideline development through external stakeholder review. P 149 | Patients involved were provided with equal status and used throughout the development process. A patient partner facilitator was used to support the panellists and provide monthly calls, guidance, and  non-financial support. |  |
| --- | --- | --- | --- | --- | --- |

UNITED KINGDOM

# National Institute for Health and Care Excellence (NICE) (UK)

|  | Q1 Who to involve? | Q2 How to recruit and  from where? | Q3 When to involve? | Q4 How does PPI  contribute? | * G-I-N Standards for Clinical Practice Guidelines. |
| --- | --- | --- | --- | --- | --- |
| Developing NICE  guidelines: the  manual [25] | Patients, service users,  carers, public including  voluntary, charitable  and community  organisations. All NICE  advisory committees  and working groups  have at least 2 lay  members. Specific  guidelines for involving  children and young | “The exact composition  of the committee is  tailored to the guideline  topic” (p. 52). The  process of recruitment  is not specified, other  than to call for  expressions of interest  for general involvement  with NICE. | Patients and public  undertake the same roles  as the health  professionals i.e.  'assessing evidence and  drafting  recommendations. All  stakeholder organisations  must be involved at key  stages: setting the scope  of the guideline, | Patients and public  undertake the same roles  as the health professionals  i.e. 'assessing evidence  and drafting  recommendations. All  stakeholder organisations  must be involved at key  stages: setting the scope  of the guideline,  circulating adverts to |  |

|  | people including those living in disadvantaged circumstances. With relevant experience of the condition and issues that matter to people with the condition. |  | circulating adverts to members for recruitment to the guideline development group, responding to calls for evidence, commenting on draft guideline. | members for recruitment to the guideline development group, responding to calls for evidence, commenting on draft guideline. |  |
| --- | --- | --- | --- | --- | --- |
| NICE's approach to  public involvement in  guidance and  standards: a practical  guide [26] | NICE uses the  following: patients,  people who use health  and care services,  carers, and the public.  The lay people and  organisations that  represent their interests  have an opportunity to  contribute in equal  value. This includes  volunteer and  community sector  organisation (national)  that are relevant to the  topic at hand. | The NICE structure  uses the Public  Involvement  Programme (PIP) to  engage all stakeholders  and participate in  guideline development.  All NICE guidelines  have two lay members  that have experience in  the conditions. Lay  members are identified  through advertising and  recruitment (roles  advertised for 4 weeks  on NICE website,  standardised questions  with no CVs, and  support information),  targeted identification  through expressions of  interests (lay people  who have previously  worked with NICE), | The NICE structure uses  the following stages of  guideline development:  Referral à Scoping à  Development à  Consultation à  Publication à  Implementation support.  Referral is used for  government departments,  stakeholders, and  voluntary and community  sectors. Notification can  use members of the  public to help identify an  issue, but it can also be  referred by clinicians or  companies. Scoping uses  stakeholder engagement.  Consultation allows for  implementation of  voluntary and community  organisations. Individuals  are allowed to comment, | The lay people could be  used in the following  ways: a core member of a  standing committee, a  topic expert member of a  standing committee, a  member of a topic-  specific committee, and as  an expert witness. This  may also include  involvement in group  discussions, interviews or  surveys outside of the lay  people to engage with the  community. |  |

|  |  | and nomination (expert witnesses are invited to give testimony) (see page 11). | but it is encouraged through stakeholders. Publication involves staff and volunteers. |  |  |
| --- | --- | --- | --- | --- | --- |
| Decision-making and mental capacity [27] | Not reported. | Not reported. | Not reported. | Not reported. | No |
| Faltering growth: recognition and management of faltering growth in children [28] | Not reported. | Not reported. | Not reported. | Not reported. | No |
| Intrapartum care for women with existing medical conditions or obstetric complications and their babies. [29] | Not reported. | Not reported. | Not reported. | Not reported. | No |
| Mental health of adults in contact with the criminal justice system [30] | Not reported. | Not reported. | Not reported. | Not reported. | No |
| Workplace health: long-term sickness absence and capability to work [31] | Not reported. | Not reported. | Not reported. | Not reported. | No |

* Effective August 2013, G-I-N requested voluntary reporting on the G-I-N Guideline Standards when a guideline is submitted for inclusion in the G-I-N Library.

References

1.	American Society of Clinical Oncology. ASCO Guidelines Methodology Manual. 2019. American Society of Clinical Oncology: Alexandria.

2.	Allison KH, Hammond MEH, Dowsett M, McKernin SE, Carey LA, Fitzgibbons PL, et al. Estrogen and Progesterone Receptor Testing in Breast Cancer: ASCO/CAP Guideline Update. J Clin Oncol. 2020; 38(12): 1346-1366.

3.	Hassett MJ, Somerfield MR, Baker ER, Cardoso F, Kansal KJ, Kwait DC, et al. Management of Male Breast Cancer: ASCO Guideline. J Clin Oncol. 2020; 38(16): 1849-1863.

4.	Konstantinopoulos PA, Norquist B, Lacchetti C, Armstrong D, Grisham RN, Goodfellow PJ, et al. Germline and Somatic Tumor Testing in Epithelial Ovarian Cancer: ASCO Guideline. J Clin Oncol. 2020; 38(11): 1222-1245.

5.	Tew WP, Lacchetti C, Ellis A, Maxian K, Banerjee S, Bookman M, et al. PARP Inhibitors in the Management of Ovarian Cancer: ASCO Guideline. J Clin Oncol. 2020; 38(30): 3468-3493.

6.	Trabulsi EJ, Rumble RB, Jadvar H, Hope T, Pomper M, Turkbey B, et al. Optimum Imaging Strategies for Advanced Prostate Cancer: ASCO Guideline. J Clin Oncol. 2020; 38(17): 1963-1996.

7.	Rosenfeld RM, Shiffman RN, and Robertson P. Clinical Practice Guideline Development Manual, Third Edition: a quality-driven approach for translating evidence into action. Otolaryngol Head Neck Surg. 2013; 148(1 Suppl): S1-55.

8.	Chandrasekhar SS, Tsai Do BS, Schwartz SR, Bontempo LJ, Faucett EA, Finestone SA, et al. Clinical Practice Guideline: Sudden Hearing Loss (Update). Otolaryngol Head Neck Surg. 2019; 161(1_suppl): S1-s45.

9.	Basura GJ, Adams ME, Monfared A, Schwartz SR, Antonelli PJ, Burkard R, et al. Clinical Practice Guideline: Ménière's Disease. Otolaryngol Head Neck Surg. 2020; 162(2_suppl): S1-s55.

10.	Rosenfeld RM, Shin JJ, Schwartz SR, Coggins R, Gagnon L, Hackell JM, et al. Clinical Practice Guideline: Otitis Media with Effusion (Update). Otolaryngol Head Neck Surg. 2016; 154(1 Suppl): S1-s41.

11.	Ishii LE, Tollefson TT, Basura GJ, Rosenfeld RM, Abramson PJ, Chaiet SR, et al. Clinical Practice Guideline: Improving Nasal Form and Function after Rhinoplasty. Otolaryngol Head Neck Surg. 2017; 156(2_suppl): S1-s30.

12.	Bhattacharyya N, Gubbels SP, Schwartz SR, Edlow JA, El-Kashlan H, Fife T, et al. Clinical Practice Guideline: Benign Paroxysmal Positional Vertigo (Update) Executive Summary. Otolaryngol Head Neck Surg. 2017; 156(3): 403-416.

13.	National Health and Medical Research Council. Procedures and requirements for meeting the 2011 NHMRC standard for clinical practice guidelines - Summary for developers. 2011. National Health and Medical Research Council: Melbourne.

14.	Cancer Council Australia. Clinical practice guidelines for keratinocyte cancer: Administrative Report 2019. Available from: https://www.cancer.org.au/assets/pdf/archived-keratinocyte-cancer-guidelines-29-May-2019. [Accessed: 25 March 2025]

15.	Cancer Council Australia. Clinical practice guidelines for the prevention, early detection and management of colorectal cancer: Administrative Report. 2017. Available from: https://www.cancer.org.au/clinical-guidelines/bowel-cancer/colorectal-cancer. [Accessed: 25 March 2025]

16.	Cancer Council Australia. Clinical practice guidelines for surveillance colonoscopy: Administrative Report. 2018. Available from: https://www.cancer.org.au/assets/pdf/archived-bowel-cancer-surveillance-colonoscopy-guidelines-3-april-2018. [Accessed: 25 March 2025]

17.	Mazza D, Brijnath, B., Chakraborty, S.P. and the Guideline Development Group,. Clinical guideline for the diagnosis and management of work-related mental health conditions in general practice – Administrative Report. . 2019. Available from: https://www.racgp.org.au/FSDEDEV/media/documents/Clinical%20Resources/Guidelines/Mental%20health/Work-related-mental-health-conditions-in-general-practice.pdf. [Accessed: 25 March 2025]

18.	Buckland D, Bashir N, Moore J, and Straus S. CTFPHC patient engagement protocol. 2017. Li Ka Shing Institute, St. Michael’s Hospital: Toronto.

19.	Groulx S, Limburg H, Doull M, Klarenbach S, Singh H, Wilson BJ, et al. Guideline on screening for esophageal adenocarcinoma in patients with chronic gastroesophageal reflux disease. Cmaj. 2020; 192(27): E768-e777.

20.	Birtwhistle R, Morissette K, Dickinson JA, Reynolds DL, Avey MT, Domingo FR, et al. Recommendation on screening adults for asymptomatic thyroid dysfunction in primary care. Canadian Medical Association Journal. 2019; 191(46): E1274-E1280.

21.	Lang E, Colquhoun H, LeBlanc JC, Riva JJ, Moore A, Traversy G, et al. Recommendation on instrument-based screening for depression during pregnancy and the postpartum period. Canadian Medical Association Journal. 2022; 194(28): E981-E989.

22.	Thériault G, Limburg H, Klarenbach S, Reynolds DL, Riva JJ, Thombs BD, et al. Recommendations on screening for primary prevention of fragility fractures. Cmaj. 2023; 195(18): E639-e649.

23.	IOM (Institute of Medicine). Clinical Practice Guidelines We Can Trust. 2011. National Academies Press Washington, DC.

24.	Srivastava A, Santagostino E, Dougall A, Kitchen S, Sutherland M, Pipe SW, et al. WFH Guidelines for the Management of Hemophilia, 3rd edition. Haemophilia. 2020; 26 Suppl 6: 1-158.

25.	National Institute for Health and Care Excellence. Developing NICE guidelines: the manual. 2014. National Institute for Health and Care Excellence: London.

26.	National Institute for Health and Care Excellence. NICE’s approach to public involvement in guidance and standards: a practical guide. 2015. National Institute for Health and Care Excellence: London.

27.	National Institute for Health and Care Excellence. Decision-making and mental capacity: NICE guideline [NG108]. 2018. Available from: https://www.nice.org.uk/guidance/NG108. [Accessed: 25 March 2025]

28.	National Institute for Health and Care Excellence. Faltering growth: recognition and management of faltering growth in children: NICE guideline [NG75]. 2017. Available from: https://www.nice.org.uk/guidance/ng75 [Accessed: 25 March 2025]

29.	National Institute for Health and Care Excellence. Intrapartum care for women with existing medical conditions or obstetric complications and their babies: NICE guideline [NG121]. 2019. Available from: https://www.nice.org.uk/guidance/ng121. [Accessed: 25 March 2025]

30.	National Institute for Health and Care Excellence. Mental health of adults in contact with the criminal justice system: NICE guideline [NG66]. 2017. Available from: https://www.nice.org.uk/guidance/ng66 [Accessed: 25 March 2025]

31.	National Institute for Health and Care Excellence. Workplace health: long-term sickness absence and capability to work: NICE guideline [NG146]. 2019. Available from: https://www.nice.org.uk/guidance/ng146. [Accessed: 25 March 2025]
